# Clinical and genetic characterization of a Taiwanese cohort with spastic paraparesis combined with cerebellar involvement

**DOI:** 10.3389/fneur.2022.1005670

**Published:** 2022-09-30

**Authors:** Min-Yu Lan, Chin-Song Lu, Shey-Lin Wu, Ying-Fa Chen, Yueh-Feng Sung, Min-Chien Tu, Yung-Yee Chang

**Affiliations:** ^1^Department of Neurology, Kaohsiung Chang Gung Memorial Hospital and Chang Gung University College of Medicine, Kaohsiung, Taiwan; ^2^Center for Parkinson's Disease, Kaohsiung Chang Gung Memorial Hospital and Chang Gung University College of Medicine, Kaohsiung, Taiwan; ^3^Center for Mitochondrial Research and Medicine, Kaohsiung Chang Gung Memorial Hospital and Chang Gung University College of Medicine, Kaohsiung, Taiwan; ^4^Professor Lu Neurological Clinic, Taoyuan, Taiwan; ^5^Department of Neurology, Landseed International Hospital, Taoyuan, Taiwan; ^6^Department of Neurology, Changhua Christian Hospital, Changhua, Taiwan; ^7^Department of Electrical Engineering, National Changhua University of Education, Changhua, Taiwan; ^8^Department of Neurology, Tri-Service General Hospital, National Defense Medical Center, Taipei, Taiwan; ^9^Department of Neurology, Taichung Tzu Chi Hospital, Buddhist Tzu Chi Medical Foundation, Taichung, Taiwan; ^10^Department of Neurology, School of Medicine, Tzu Chi University, Hualien, Taiwan

**Keywords:** hereditary spastic paraplegia, spinocerebellar ataxia, whole-exome sequencing, thin corpus callosum, ataxia-spasticity spectrum diseases

## Abstract

Hereditary spastic paraplegias (HSPs) are a heterogeneous group of neurodegenerative disorders clinically characterized by progressive lower-limb spasticity. Cerebellar ataxia commonly co-occurs with complicated HSPs. HSP with concurrent cerebellar ataxia has significant clinical and genetic overlaps with hereditary cerebellar ataxia (HCA) and other inherited neurological diseases, adding to the challenge of planning genetic testing for the disease. In this study, we characterized clinical features of a cohort of 24 patients (male/female: 15/9) from 22 families who presented spastic paraparesis combined with cerebellar involvement, with a median disease onset age 20.5 (range 5–53) years. Aside from the core phenotype, 18 (75%) patients had additional neuropsychiatric and systemic manifestations. A stepwise genetic testing strategy stratified by mode of inheritance, distinct neuroimaging features (e.g., thin corpus callosum), population-specific prevalence and whole-exome sequencing was utilized to investigate the genetic etiology. Causative mutations in up to 10 genes traditionally related to HSP, HCA and other neurogenetic diseases (autosomal recessive spastic ataxia of Charlevoix-Saguenay, neurodegeneration with brain iron accumulation, and progressive encephalopathy with brain atrophy and thin corpus callosum) were detected in 16 (73%) of the 22 pedigrees. Our study revealed the genetic complexity of HSP combined with cerebellar involvement. In contrast to the marked genetic diversity, the functions of the causative genes are restricted to a limited number of physiological themes. The functional overlap might reflect common underlying pathogenic mechanisms, to which the corticospinal tract and cerebellar neuron circuits may be especially vulnerable.

## Introduction

Hereditary spastic paraplegias (HSPs) comprise a heterogeneous group of inherited neurodegenerative diseases with the common clinical characteristics of progressive spasticity and weakness of the lower extremities. Pathophysiologically, HSP is a primary distal axonopathy that affects both the corticospinal and posterior column sensory tracts, with maximal degeneration at the end of the nerve fibers in these tracts. The genetic basis of HSP is also complex. To date, there have been 87 HSP genes or loci registered in Online Mendelian Inheritance of Men (1), suggesting diverse molecular pathogenetic mechanisms underlying this group of diseases. All modes of inheritance have been observed in HSPs. HSP is classified as a pure or complicated form according to the absence or presence of additional neurological or systemic features, such as cognitive impairment, epilepsy, cerebellar ataxia, thin corpus callosum, peripheral neuropathy, optic neuropathy, retinopathy, and deafness (2, 3). Pure forms of HSP tend to be inherited in an autosomal dominant (AD) pattern, whereas complicated HSPs are more frequently autosomal recessive (AR).

Cerebellar ataxia is a common concurrent neurological manifestation of complicated HSPs and is present in more than 30 disease entities and ~30% of complicated HSP patients (4–6). Conversely, hereditary cerebellar ataxia (HCA) may occasionally present with severe lower limb spasticity in the early stage of the disease, resembling HSP until the ataxic component becomes more prominent (7). Moreover, HSP has significant clinical and genetic overlaps with other categories of neurodegenerative disease, such as HCA, spastic ataxia (SPAX), genetic leukoencephalopathy, motor neuron disease and inherited neuropathy (7, 8). Because of the individual rarity, phenotype overlaps and genetic heterogeneity of the conditions, the molecular diagnosis of HSP and “HSP mimics” remains challenging.

In this study, we conducted a clinical and genetic characterization study in a cohort of Taiwanese patients whose main clinical presentation was spastic paraparesis combined with cerebellar dysfunction. Through a strategy incorporating inheritance and brain imaging-guided target gene testing, followed by screening for prevalent diseases and whole-exome sequencing (WES), genetic diagnosis was achieved in more than two-thirds of cases. In addition to demonstrating the effectiveness of customized genetic testing in the diagnosis of HSP combined with cerebellar involvement, this study provides insights into the genetic heterogeneity of neurodegenerative diseases with a phenotype of coexisting spasticity and ataxia.

## Methods

### Participants

A total of 30 patients of complicated HSP from 27 kindreds were recruited at or referred to Kaohsiung Chang Gung Memorial Hospital, Taiwan, between January 2015 and June 2021. Among them, 24 patients from 22 families had combined cerebellar involvement in addition to an HSP-like core phenotype. The clinical diagnosis of HSP was based on the following: (i) clinically spastic paraplegia as an early and prominent sign of a degenerative disease affecting the nervous system, or spastic tetraparesis with earlier and more severe affliction of the lower limbs; and (ii) no acquired causes of the presenting symptoms (9). Cerebellar involvement was defined as cerebellar ataxia demonstrated by neurological examination, or as atrophy of the cerebellum more than other brain regions on magnetic resonance imaging (MRI) when ataxia could not be demonstrated by neurological examination due to severe weakness or spasticity. Clinical information and brain MRI findings were collected. Ambulatory disability was scored on a six-level scale: 0 = asymptomatic, 1 = minimally impaired (abnormal gait but able to run), 2 = mildly impaired (able to walk independently but not run), 3 = moderately impaired (able to walk with an aid), 4 = severely impaired (wheelchair bound), and 5 = confined to bed. The study was approved by the Chang Gung Memorial Hospital Institutional Review Board (No. 100-4460C). Written informed consent was obtained from participants or, in the case of minors, from a parent.

### Genetic study strategy

**Figure 1:**
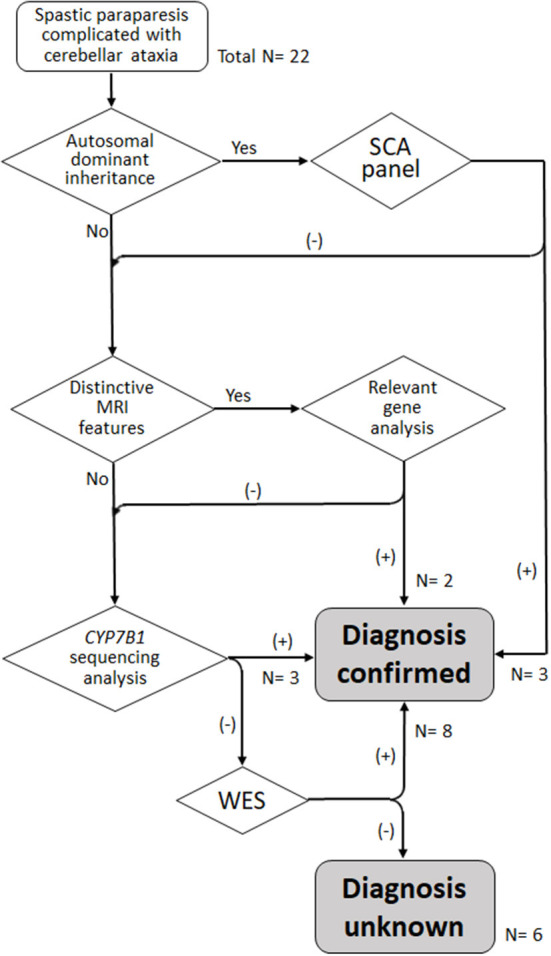
Algorithm for stratified genetic testing in this study. *N*, number of index cases.

To focus the analysis on the most phenotype-relevant genes, reducing cost and time consumed and ensuring diagnostic yield at the same time, a stepwise genetic testing strategy was applied (Figure 1). In the first step, the mode of inheritance was considered. AD inheritance was defined as the presence of affected individuals in at least two successive generations in a family. The inheritance pattern was classified as AR if there were at least two affected siblings in a generation without the presence of affected members in two successive generations. First, the patients with AD inheritance were screened with a panel of spinocerebellar ataxia (SCA) genes, including SCA1 (*ATXN1*), SCA2 (*ATXN2*), SCA3 (*ATXN3*), SCA6 (*CACNA1A*), SCA7 (*ATXN7*), SCA17 (*TBP*) and dentatorubropallidoluysian atrophy (*ATN1*). In the second stage, we observed characteristic brain MRI features, such as thin corpus callosum (TCC), which might hint at probable HSP subtypes and thus guide sequencing analysis of the most relevant genes. Third, because SPG5 is the most common complicated form of HSP in Taiwan, attributed to a founder *CYP7B1* mutation, c.334C>T (p.Arg112Ter), and usually combined with cerebellar ataxia (10, 11), genetic testing of the gene *CYP7B1* was performed for the cases that tested negative in MRI-directed genetic testing and for those without specific brain MRI findings. Finally, WES was conducted for the remaining undiagnosed cases.

### Exome sequencing, bioinformatic analysis and determination of pathogenic variants

A SureSelect Human All Exon 50 Mb Kit (Agilent Technologies) was used to capture the exome library, and sequencing was performed on an Illumina HiSeq2000 (Illumina). Approximately 52 million paired reads were mapped to the human reference genome. Burrows-Wheeler Aligner and Genome Analysis Toolkit were used for read mapping and variant calling. A coverage analysis revealed that 90% of the target regions were covered by ≥ 35 reads.

To determine causative variants, we prioritized WES variants with the following process. First, variants in genes that were included in the lists of HSP/HSP-like genes, HCA genes and genetic leukoencephalopathy genes (Supplementary material 1) or those that were novel or coded as “pathogenic” or “likely pathogenic” according to ClinVar and related to a relevant neurological disease were selected. Second, only variants with a predicted functional impact on coding regions (e.g., missense, nonsense, insertion, deletion or splicing site variation) were considered. Third, based on the Genome Aggregation Database (https://gnomad.broadinstitute.org/), Exome Variant Server (https://evs.gs.washington.edu/EVS/), and Taiwan Biobank (https://taiwanview.twbiobank.org.tw/index), variants with frequencies >0.1% in any population were discarded. Finally, mode of inheritance and clinical data were taken into account to evaluate the relevance of the variants to the phenotypes.

The impact of a missense variant on encoded protein structure or function was predicted by SIFT (https://sift.bii.a-star.edu.sg/), PROVEAN (http://provean.jcvi.org/index.php), and CADD (https://cadd.gs.washington.edu/). The impact of splicing site variants on RNA transcription was interpreted with the Spice Site Prediction by Neural Network (https://www.fruitfly.org/seq_tools/splice.html). Variants that were interpreted as “pathogenic” or “likely pathogenic” based on the American College of Medical Genetics and Genomics and Association of Molecular Pathology (ACMG-AMP) guidelines (12) were considered to be significantly pathogenic unless otherwise specified. The novelty of the pathogenic variant was checked with the Human Gene Mutation Database (http://www.hgmd.cf.ac.uk/ac/index.php) and ClinVar (https://www.ncbi.nlm.nih.gov/clinvar/). All reported variants were confirmed by Sanger sequencing. The parental origin of the detected variants was investigated by sequencing the genes in each proband's parents and siblings where available.

## Results

There were 24 patients (male/female: 15/9) from 22 families, including four cases from four AD families (D1–D4), six cases from four AR families (R1–R4), and 14 sporadic cases (S1–S14) (Table 1). The median age was 36 (range 13–65) years at the time of examination, and the median disease onset age was 20.5 (range 5–53) years. Twenty patients presented both cerebellar ataxia in neurological examination and cerebellar atrophy on MRI, one patient presented cerebellar ataxia in neurological examination with normal brain MRI (S12), and three patients (R2, R3-2 and S10) showed cerebellar atrophy on MRI (Supplementary material 2) while ataxia could not be checked by neurological examination due to severe weakness or spasticity. Aside from the core feature of spastic paraparesis combined with cerebellar ataxia or atrophy, 18 (75%) patients had additional neuropsychiatric manifestations, including cognitive impairment (*n* = 8), parkinsonism or other extrapyramidal signs (dystonia, myoclonus, or tremor) (*n* = 8), polyneuropathy (*n* = 6), ophthalmoplegia (*n* = 4), intellectual disability (*n* = 1), optic atrophy (*n* = 1) and bipolar disorder (*n* = 1). For systemic comorbidity, one patient (R1-2) had rheumatoid arthritis, and another had insulin-dependent diabetes mellitus (S9).

**Table 1:** Demographic data and clinical features of cases in the study cohort.

**Case**	**Sex**	**Age of onset (years)**	**Age of examination (years)**	Ambulatory function*	**Additional clinical features**	**Special brain MRI findings**
D1	F	10	13	2	Dystonia	
D2	M	50	55	2	Ophthalmoplegia, parkinsonism	
D3	M	43	46	2	Ophthalmoplegia, polyneuropathy, parkinsonism	
D4	M	31	32	1	Ophthalmoplegia, sensory ataxia, parkinsonism	
R1-1	M	20	38	4	Cognitive impairment	
R1-2	F	21	42	4	Cognitive impairment, rheumatoid arthritis	
R2	F	23	51	4	Cognitive impairment, polyneuropathy	Abnormal brainstem signals, thin corpus callosum
R3-1	M	17	27	2	Cognitive impairment, bipolar disorder, parkinsonism	
R3-2	F	37	38	2	Cognitive impairment, parkinsonism, dystonia	
R4	F	42	45	2		Calcification of medial globus pallidus, lacune in left putamen
S1	F	11	28	4		Leukoencephalopathy
S2	M	33	42	3	Intellectual disability, delayed development, optic atrophy	Thin corpus callosum
S3	M	10	16	2		
S4	M	12	34	2		Leukoencephalopathy
S5	F	33	41	2	Ophthalmoplegia	Leukoencephalopathy
S6	M	23	32	3	Cataract, cognitive impairment, polyneuropathy	
S7	M	34	42	4	Cognitive impairment, myoclonus	
S8	M	14	24	3	Isometric tremor	
S9	M	20	22	2	Polyneuropathy, diabetes mellitus	Leukoencephalopathy
S10	M	5	19	2	Polyneuropathy	
S11	F	13	27	2		
S12	M	45	49	2		
S13	F	13	20	2	Cognitive impairment	Thin corpus callosum, leukoencephalopathy
S14	M	53	65	3	Polyneuropathy	

D, autosomal dominant inheritance; F, woman; M, man; R, autosomal recessive inheritance; S, sporadic.

*0 asymptomatic, 1 abnormal gait but able to run, 2 able to walk independently but not run, 3 able to walk with an aid, 4 wheelchair bound, 5 confined to bed.

**Figure 2:**
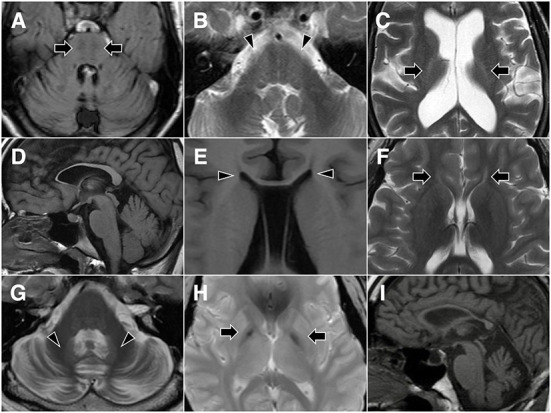
Representative brain MR images. Patient R2 shows horizontal hypointensity strips on FLAIR imaging (**A**, arrows), along with a hyperintense zone in the lateral pons on T2-weighted imaging (**B**, arrowheads) and a hyperintense peri-thalamic rim (**C**, arrows). Patient S13 shows thin corpus callosum **(D)** and a box-shaped calloso-caudate angle (**E**, arrowheads) on T1-weighted imaging, as well as hyperintense streaks in the forceps minor of the corpus callosum (“ears of the lynx” sign) on T2-weighted imaging (**F**, arrows). Patient D4 demonstrates cerebellar atrophy and T2 hyperintensity of the dentate nucleus (**G**, arrowheads), as well as T2 gradient-echo hypointensity of the medial globus pallidus (**H**, arrows). Midsagittal T1-weighted imaging of patient S2 shows thin corpus callosum and cerebellar atrophy **(I)**.

The first tier of genetic testing was the SCA gene panel for the four patients with a family history of autosomal dominant inheritance, and it revealed pathogenic CAG repeat expansion in *ATXN3* in three of them (15/83 in D1, 15/67 in D2, and 20/72 in D3). Then, relevant genes were tested in three patients with characteristic brain MRI features that suggested specific disease entities. For patient R2, brain MRI showed horizontal hypointensity strips, a hyperintensity zone in the lateral pons, a hyperintense peri-thalamic rim on T2W and FLAIR imaging, and TCC (Figures 2A–C). These findings were typical of autosomal recessive spastic ataxia of Charlevoix-Saguenay (ARSACS) (13). Sequencing analysis of *SACS* revealed a homozygous frameshift variant, c.8621_8624 del (p.Ser2874TrpfsTer), which confirmed the diagnosis. Brain MRI of patients S2 and S13 showed TCC. Patient S13 also presented T2-hyperintense streaks in the forceps minor of the corpus callosum (“ears of the lynx” sign) (14) and a box-shaped calloso-caudate angle (Figures 2D–F). For the two cases, sequencing analysis for genes of the three HSPs most frequently associated with TCC, *SPG11, ZFYVE26* (SPG15 gene) and *SPG21*, was performed. SPG11 was confirmed for patient S13 with the detection of two novel heterozygous variants, c.1602+1 G>C and c.3175_3176 delinsTG (p.Ala1059Ter), in *SPG11*, while no pathogenic variant was detected in the three genes in patient S2. Afterward, sequencing analysis of *CYP7B1* was performed in the remaining probands, and SPG5 was confirmed in three cases (S3, S4 and S12). All three SPG5 cases carried the *CYP7B1* variant c.334 C>T (p.Arg112Ter), which was homozygous in patients S3 and S12 and was heterozygous and combined with a missense variant c.1316 T>G (p.Leu439Arg) in patient S4. Although the variant c.1316 T>G was categorized as a “variant of uncertain significance” based on the ACMG-AMP criteria, it has been reported previously in another Taiwanese SPG5 patient (11).

WES was then performed in the remaining patients who tested negative in the previous genetic analysis. Significant variants in the spasticity or ataxia-relevant genes were detected in all of the one AD and 3 AR cases and 4 of the remaining 10 sporadic cases. A novel heterozygous variant c.101A>G (p.Asn34Ser) in *STUB1*, a gene linked to SCA48, was detected in Patient D4. The patient's father had a phenotype of slowly progressive pure cerebellar dysfunction and carried the same *STUB1* variant (see Case reports). The R1 proband (R1-1) had two heterozygous variants in *GBA2*, including c.2618 G>A (p.Arg873His), a previously reported disease-causing variant (15), and a novel mutation c.2635 C>T (p.Arg879Trp), and SPG46 was diagnosed. The R3 proband (R3-1) was heterozygous for two reported variants, c.991 G>T (p.Asp331Tyr) and c.1427+1 G>A in *PLA2G6* (16, 17), and *PLA2G6*-associated neurodegeneration (PLAN) was diagnosed. The same *GBA2* and *PLA2G6* variants were also detected in the affected siblings of the two probands. Pathogenic variants in *CAPN1* (SPG76 gene) were detected in two patients, including a homozygous novel nonsense variant c. 894 G>A (p.Trp298Ter) in patient R4 and two previously reported heterozygous variants, c.188 dup (p.Val64GlyfsTer) and c.1493 C>T (p.Pro498Leu) (18, 19), in patient S11. Patient S2 had a variant c.1340 C>T (p.Ala447Val) (“likely pathogenic” registered in ClinVar) and a previously reported variant c.3365 C>T (p.Pro1122Leu) (20) in *TBCD*, a gene linked to progressive encephalopathy with brain atrophy and thin corpus callosum (PEBAT, OMIM:617193). Two heterozygous missense variants in *KIF1A* (SPG30 gene) were detected in two patients, including a novel variant c.1031 C>T (p.Thr344Met) in patients S6 and a previously reported variant c.761 G>A (p.Arg254Gln) (21) in patient S10. *De novo* mutations were considered for both patients, as the corresponding *KIF1A* variants were not detected in their parents. In all, genetic mutation was detected in 16 (73%) of the 22 probands of this cohort (Figure 1; Table 2).

**Table 2:** Causative and likely causative genetic variants identified in the study cohort.

**Case**	**Gene**	**Variants**	**Missense variant**			**NNSplice**	**ACMG-AMP criteria**	**Disease**
			**SIFT**	**PROVEAN**	**CADD**			
D1	*ATXN3*	CAG repeats 15/83	—	—	—	—	—	SCA3
D2	*ATXN3*	CAG repeats 15/67	—	—	—	—	—	SCA3
D3	*ATXN3*	CAG repeats 20/72	—	—	—	—	—	SCA3
D4	*STUB1*	c.101A>G (p.Asn34Ser)*	0.005	−4.06	26.9	—	Likely pathogenic (PM1, PM2, PP1, PP2, PP3)	SCA48
R1	*GBA2*	c.2618G>A (p.Arg873His)	0	−4.73	29.0	—	Likely pathogenic (PM2, PM3, PP3, PP5)	SPG46
		c.2635C>T (p.Arg879Trp)*	0	−7.55	29.1	—	Likely pathogenic (PM2, PM3, PM5, PP3)	
R2	*SACS*	c.8621_8624 del (p.Ser2874TrpfsTer), homozygous	—	—	—	—	Pathogenic (PVS1, PM2, PP5)	ARSACS
R3	*PLA2G6*	c.991G>T (p.Asp331Tyr)	0.003	−4.43	26.0	—	Pathogenic (PS3, PM2, PM3, PP2, PP3)	PLAN
		c.1427+1G>A	—	—	24.3	< 0.1	Pathogenic (PVS1, PM2, PM3, PP5)	
R4	*CAPN1*	c.894G>A (p.Trp298Ter)*, homozygous	—	—	39.0	—	Likely pathogenic (PVS1, PM2)	SPG76
S2	*TBCD*	c.1340C>T (p.Ala447Val)	0.002	−3.68	24.5	—	Likely pathogenic (PM2, PM3, PP3, PP5)	PEBAT
		c.3365C>T (p.Pro1122Leu)	0.118	−6.77	24.5	—	Pathogenic (PS3, PM2, PM3, PP3, PP5)	
S3	*CYP7B1*	c.334C>T (p.Arg112Ter), homozygous	—	—	23.9	—	Pathogenic (PVS1, PM2, PP5)	SPG5
S4	*CYP7B1*	c.334C>T (p.Arg112Ter)	—	—	23.9	—	Pathogenic (PVS1, PM2, PP5)	SPG5
		c.1316T>G (p.Leu439Arg)	0.145	−3.49	15.08	—	VUS (PM2, PP5)	
S6	*KIF1A*	c.1031C>T (p.Thr344Met)^*^^#^	0	−5.69	25.7	—	Likely pathogenic (PM1, PM2, PM6, PP3, PP5)	SPG30
S10	*KIF1A*	c.761G>A (p.Arg254Gln)^#^	0.001	−3.47	27.1	—	Likely pathogenic (PM1, PM2, PM5, PM6, PP3, PP5)	SPG30
S11	*CAPN1*	c.188dup (p.Val64GlyfsTer)	—	—	—	—	Pathogenic (PVS1, PM2, PM3, PP5)	SPG76
		c.1493C>T (p.Pro498Leu)	0.011	−9.07	23.8	—	Likely pathogenic (PM1, PM2, PM3, PP2, PP3)	
S12	*CYP7B1*	c.334C>T (p.Arg112Ter), homozygous	—	—	23.9	—	Pathogenic (PVS1, PM2, PP5)	SPG5
S13	*SPG11*	c.1602+1G>C*	—	—	27.7	< 0.1	Likely pathogenic (PVS1, PM2)	SPG11
		c.3175_3176 delinsTG (p.Ala1059Ter)*	—	—	—	—	Likely pathogenic (PVS1, PM2)	

—, not applicable.

*Novel mutation.

^#^*De novo* mutation.

ARSACS, autosomal recessive spastic ataxia of Charlevoix-Saguenay; D, autosomal dominant inheritance; NNSplice, Splice Site Prediction by Neural Network; PEBAT, progressive encephalopathy with brain atrophy and thin corpus callosum; PLAN, PLA2G6-associated neurodegeneration; R, autosomal recessive inheritance; S, sporadic; VUS, variant of uncertain significance.

### Case reports

#### Patient D4

The 32-year-old security guard had an unsteady gait that had developed approximately one-half to 1 year prior. Meanwhile, slurred speech, hand awkwardness and slower general movements developed insidiously during the period. Neurological examination revealed normal cognitive function (a Mini-Mental State Examination score of 29), combined nuclear and supranuclear ophthalmoplegia, dysmetric saccades, increased muscle tone in the lower limbs, general hyperreflexia, four limb and trunk ataxia, a positive Romberg test, and a mixed ataxic and spastic gait. Brain MRI showed diffuse cerebellar atrophy, T2 hyperintensity of the dentate nucleus, and T2 gradient-echo hypointensity of the medial globus pallidus (Figures 2G,H). The peripheral nerve conduction study was normal. WES detected a heterozygous missense variant, c.101A>G (p.Asn34Ser), in *STUB1*.

The patient's father, 63 years old, had unsteady gait with slow progression in severity since the early 40 years of age. Unlike the proband, he presented with lower-limb- and trunk-predominant cerebellar ataxia without ophthalmoplegia or spasticity. Brain MRI showed pancerebellar atrophy, and genetic testing revealed heterozygosity for the same *STUB1* variant. The proband's mother and siblings were normal in neurological examination and did not carry the *STUB1* variant (Supplementary material 3). The intrafamilial segregation of the phenotype and the genetic variant was consistent with AD inheritance, and thus SCA48 was diagnosed.

#### Patient R3-1

The proband, a 37-year-old male, had unsteady gait since 20 years ago. He had intellectual disability and delayed developmental milestones and received integrated rehabilitation from 2 to 5 years of age. He had developed psychiatric symptoms including delusions, visual hallucinations and emotional lability in his early 20s and had been diagnosed with bipolar disorder. Slow movements, dysuria and urine incontinence appeared along with progression of gait disturbance in the following few years. Neurological examination showed weakness of the distal four limbs, increased lower limb muscle tone, general hyperreflexia, bilateral extensor plantar reflex and a right spastic gait, apart from cognitive impairment, ophthalmoplegia, hypophonic dysarthria, four limb dysmetria and general hypokinesia. Brain MRI showed marked pancerebellar atrophy. WES detected compound heterozygous variants, c.991 G>T (p.Asp331Tyr) and c.1427+1 G>A, in *PLA2G6*, and PLAN was diagnosed accordingly.

His 39-year-old sister (R3-2) had a much later disease-onset age. She presented with rapid progression of motor and cognitive symptoms since the onset 2 years ago. Neurological examination showed general hyperreflexia, bradykinesia, rigidity and dystonia as well as a spastic gait. Brain MRI showed diffuse cerebellar atrophy. Genetic testing revealed identical compound heterozygous variants in *PLA2G6*.

#### Patient S2

The 42-year-old man had intellectual disability since early childhood and delayed early development (able to stand at 24 months and walk at 30 months). Intrauterine or perinatal events were denied by his parents. Unsteady gait was noted at 33 years of age at first, and it became more severe with time and led to repeated falls. Family history was not contributory. Neurological examination revealed impaired cognitive function, optic atrophy, strabismus, increase in lower limb muscle tone, muscle atrophy at distal limbs, general hyperreflexia and right side extensor plantar reflex, four limb and trunk ataxia and bilateral spastic gait. A nerve conduction study showed axonal polyneuropathy in the lower limbs. Brain MRI was remarkable for a thin corpus callosum (Figure 2I) and pancerebellar atrophy. WES detected compound heterozygous variants c.1340 C>T (p.Ala447Val) and c.3365 C>T (p.Pro1122Leu) in *TBCD*.

#### Patient S12

The 49-year-old male born to consanguineous (3rd degree) parents had insidious development of gait abnormality that progressed gradually in the past 5 years. He felt stiffness of the leg muscles and had difficulty walking fast and easy tripping. Speech, swallowing and upper limb functions were not affected. Symptoms of bladder control were denied. Neurological examination showed normal cognitive function, cranial nerves and muscle bulk and strength of the four limbs. Marked muscle tone increase in the lower limbs, general hyperreflexia, bilateral ankle clonus with extensor plantar reflex, prominent impairment of posterior column sensations at distal lower limbs, lower limb and trunk ataxia, Romberg's sign, and a mixed spastic and wide-based gait pattern were noted. Sequence analysis for *CYP7B1* revealed homozygosity for c.334 C>T (p.Arg112Ter) and SPG5 was confirmed.

## Discussion

With a genetic testing strategy stratified by mode of inheritance, characteristic neuroimaging features, population-specific prevalence and WES, we detected causative mutations in disease-relevant genes in 73% (16/22) of the index cases of combined spastic paraparesis and cerebellar ataxia. In addition, half of the cases with molecular diagnosis could be confirmed before implementation of WES. It has been demonstrated that by integrating mode of inheritance and pertinent clinical data, disease-related genetic testing followed by multigene panels may improve diagnostic yield for HSP and HCA (22). Furthermore, this and our study suggest that the integrated testing flowchart not only helps to guide a time and cost-efficient molecular testing in the new generation sequencing era, but also facilitates genotype-phenotype correlation in diagnosing the heterogeneous neurogenetic disorders.

HSP and HCA are traditionally designed as two disparate groups of inherited neurodegenerative disorders. With the main clinical manifestations related to degeneration of the corticospinal tract and the cerebellar circuit, each of them encompasses an ever-expanding list of causative genes. However, with the progress of next generation sequencing and genomic studies in the past decade, genetic overlap between HSP and HCA has been increasingly recognized. In the HCA cohorts with exclusion of repeat-expansion mutations, exome-based genetic studies revealed that mutations in HSP (SPG) genes represented ~20% (ranging from 18.5 to 30%) of cases with molecular diagnosis (23–27). In fact, some genes have been linked to both HSP and HCA loci. For instance, *PNPLA6* has been identified as a cause of complicated HSP combined with motor neuropathy (SPG39) (28), and it has also been linked to AR cerebellar ataxia (29). These discoveries led the concept of a phenotype spectrum, with spasticity at one end and ataxia at the other, rather than considering HSP and HCA separately as two groups of diseases. Moreover, it is also the case that the phenotype of combined spasticity and ataxia is related to genes that classically belong to other disease categories, such as leukodystrophy, familial Parkinson's disease, frontotemporal dementia, and neurodegeneration with brain iron accumulation (NBIA) (30). These diseases have been more frequently considered jointly as “ataxia-spasticity spectrum diseases (ASSD)” (31) in replacement of the conventional but overlapping disease classifying systems.

Despite a small study cohort, causative mutation in up to 10 genes related to various neurogenetic diseases were detected in this study, indicating that etiologies are remarkably heterogeneous for spastic paraparesis combined with cerebellar ataxia. First, SCA3 was diagnosed in three cases, and SCA48 was diagnosed in one patient, supporting that HCA and HSP lie in two ends of the broad continuum of ASSD. SCAs may present predominantly with spastic paraparesis at the early stage of the disease. This distinct phenotype is more frequently observed in SCA3 cases of eastern Asian origin who carry greatly expanded *ATXN3* trinucleotide repeats (32). *STUB1* encodes CHIP, a protein that acts as a ubiquitin ligase and cochaperone and is highly expressed in the brain, especially in Purkinje cells (33). *STUB1* mutations could be presented with HCA of AR (SCAR16) and AD (SCA48) inheritance. The variant detected in this study, c.101 A>G (p.Asn34Ser), is predicted to be deleterious to the functions or structure of the encoded protein by *in silico* analysis and cosegregated with affected individuals compatible with an AD inherited mode in the reported kindred. The amino acid residue altered by this variant is located in the tetratricopeptide repeat domain, which interacts with heat shock proteins and guides them to refold misfolded proteins (34), suggesting its pathogenic role. In addition, two variants affecting the neighboring residues, p.Gly33Ser and p.Arg35Ser, have been linked to SCA48 and SCAR16, respectively (35, 36).

Five diseases belonging to HSP loci were detected, including SPG5, SPG11, SPG30, SPG46, and SPG76. SPG5 is the most common AR HSP in Taiwan and is attributed to a founder *CYP7B1* mutation, c.334C>T (p.Arg112Ter) (10, 11). All three SPG5 patients discovered in this study carried the ethnicity-specific *CYP7B1* variant. The phenotype of SPG5 related to this variant is characterized by remarkable dorsal column sensory deficits and may present with spastic paraparesis and combined cerebellar and sensory ataxia (10), as in our patients. The *CYP7B1* gene encodes the enzyme cytochrome P450 oxysterol 7α-hydroxylase, which is widely expressed in human tissues and acts on hydroxylating endogenous steroids, including the two neurosteroids dehydroepiandrosterone and pregnelonone (37). The two SPG30 patients discovered in this study were associated with *de novo* mutations of *KIF1A*. Apart from cerebellar ataxia, the two cases had additional neurological or systemic features, including polyneuropathy, cataract and cognitive decline. *KIF1A* mutations underlie a wide spectrum of neurodegenerative disorders, broadly termed *KIF1A*-associated neurological diseases (38). Among them, SPG30 could have a pure or complicated HSP phenotype and could be caused by *de novo KIF1A* mutations or inherited with an AD or AR mode (39). The *KIF1A* gene encodes a neuron-specific kinesin protein called kinesin 1a, which governs anterograde axonal transport of synaptic vesicle precursors (40). Regardless of phenotype, most of the reported cases with monoallelic mutation were related to missense variants that affect the kinesin motor domain (amino acid residues 1–365) and disrupt its binding with microtubules to process transport (41). The cohort also included two SPG46 patients from an AR family with compound *GBA2* mutations. *GBA2* encodes β-glucosidase 2, an enzyme catalyzing the cleavage of sphingolipid glucosylceramide. One of the detected mutations, c.2618 G>A (p.Arg873His), has been shown to be associated with a decrease in β-glucosidase 2 activity (42). *GBA2* mutations have been reported in patients with HSP and HCA with spasticity (15, 43). Additional clinical features may include cognitive impairment, axonal neuropathy and hearing loss. SPG76 was confirmed in AR and sporadic patients caused by *CAPN1* mutations. *CAPN1* encodes calpain-1, a calcium-dependent proteinase highly expressed in cerebellar neurons that is involved in several neurobiological functions, such as synaptic plasticity, axonal maintenance and neuronal migration and maintenance (18, 44). *CAPN1* mutations were first linked to a spastic ataxia phenotype in 2016. Since that time, there have been a significant number of SPG76 cases of east Asian origin reported in the literature (19, 45–49).

Three different diseases presenting with TCC, including SPG11, PEBAT and ARSACS, were detected in the study cohort. HSP combined with TCC is characterized to belong to a group of distinctive HSP subtypes (50). For this special HSP group, SPG11 is the common subtype, accounting for 33 and 41% of the cases in the genetic investigations of two large cohorts (51, 52). Aside from TCC, abnormality of the forceps minor of the corpus callosum may be present (14). These characteristic brain imaging features guided us to achieve diagnosis in a patient by phenotype-directed gene analysis. Clinically, SPG11 is typically associated with complicated phenotypes, including intellectual disability, progressive cognitive decline, axonal neuropathy and cerebellar signs (51, 52). Notably, we detected compound heterozygous *TBCD* mutations in the other HSP patient combined with TCC, adding a novel member in this distinctive HSP subtype. *TBCD* mutations were first linked to a rare early-onset neurodevelopmental disease (20, 53, 54), which was later termed PEBAT, in 2016. Patients with the disease typically present with copious neurodegenerative features, including microcephaly, developmental delay, intellectual disability, seizure, optic atrophy, spastic quadriparesis, ataxia and prominent brain imaging abnormalities such as brain atrophy, hypomyelination and TCC. *TBCD* encodes tubulin folding cofactor D, a tubulin-specific chaperone required for the formation of α- and β-tubulin heterodimers. Reduced TBCD levels or defective β-tubulin binding to mutant TBCD proteins results in decrease of soluble α/β tubulin levels and accelerates microtubule polymerization (20), suggesting that altered microtubule dynamics underlie the pathogenic mechanism for PEBAT.

Aside from PEBAT, two diseases not traditionally referred to as HSP, including ARSACS and PLAN, were also found in this cohort. ARSACS was first reported as a prevalent, early-onset neurodegenerative disease in Quebec, Canada, with rather homogenous clinical features, including spasticity, ataxia, optic nerve hypermyelination and intellectual disability (55). Cases of ARSACS discovered in other regions of the world may present with different phenotypes, such as later onset of disease or absence of spasticity (56). Despite the variations in clinical phenotypes, brain MR images showed distinctive features, including horizontal hypointensity in a bulky pons, longitudinal hyperintensity in the lateral pons, TCC, hyperintensity in the lateral thalamic rim on T2 images, and upper-vermis-predominant cerebellar atrophy, which serve as imaging hallmarks for ARSACS (13). PLAN is linked to *PLA2G6* mutations and belongs to NBIA, a heterogeneous group of neurodegenerative diseases characterized by iron accumulation in the basal ganglia and substantia nigra due to various disrupted cellular mechanisms (57). *PLA2G6* encodes calcium-dependent phospholipase A2, which catalyzes the hydrolysis of glycerophospholipids. PLAN comprises a continuum of distinctive diseases with overlapping clinical features (58). Patients with classical infantile neuroaxonal dystrophy and atypical neuroaxonal dystrophy present progressive neurocognitive regression during the infantile period to early childhood and axonal spheroids secondary to deranged cellular membranes in the central and peripheral nervous systems. PLA2G6-related dystonia-parkinsonism, on the other hand, typically has an onset of psychiatric symptoms, cognitive decline, pyramidal, extrapyramidal, and cerebellar features in young adulthood. Rarely, PLAN may present with early- or adult-onset spastic paraparesis combined with cerebellar ataxia, intellectual disability or Parkinsonism (59–61), adding complicated HSP to the PLAN phenotype spectrum. Intrafamilial variability of phenotype for PLA2G6-assocaited neurodegeneration has been reported previously (62). The exact mechanisms are not yet known although it is suggested that differences of genetic variants in other genes linked to neurodegenerative diseases between the affected siblings may contribute to the phenotypic heterogeneity.

A total of 10 different diseases caused by mutations in the corresponding genes were detected in this study. In contrast to the broad spectrum of genetically defined disorders for the investigated phenotype, the functions of these causative genes may fall within a relatively small number of common physiological themes at the cellular level, including microtubule maintenance (*TBCD, SACS*), axonal transport (*KIF1A*), phospholipid and fatty acid metabolism (*CYP7B1, GBA2, PLA2G6*), the endosome-lysosome-autophagosome system (*ATXN3, STUB1, SPG11, SCAS*), myelination (*GBA2*) and mitochondrial functions (*ATXN3, SACS*) (56, 63–66). Except that, endoplasmic reticulum and intracellular membrane trafficking are also associated with some genes that are involved in ASSD (e.g., *KIF1C, VAMP1*) (31), although genetic mutation of this category was not detected in this study. The functional overlap underlying the numerous ASSDs might reflect common underlying pathogenic mechanisms, to which the corticospinal tract and cerebellar neuron circuits are especially vulnerable. This pathogenicity-driven approach might not only inspire a novel mechanism-based disease classification system but also trigger pathway-targeting disease exploration and therapeutic strategies (30).

Several limitations of the study need to be addressed. First, this study was based on a relatively small cohort collected from single medical center in Taiwan. Diagnostic yield of the stratified genetic testing strategy as well as genetic spectrums for the phenotype of combined spastic paraparesis and cerebellar ataxia need to be checked by nationwide surveys with large study cohorts. Second, some patients were included in the study based on the observation of prominent cerebellar atrophy on MRI. However, brain volumetry compared with normal controls was not performed to ascertain significant decrease of cerebellar volume in these patients. Third, genetic testing methods used in this study cannot detect variants located in deep intron or exon deletion or duplication. To fill the diagnostic gap, the stepwise genetic testing protocol must be complemented with long-read whole genome sequencing for the last tier of test. Fourth, a cross-sectional data of a six-level ambulatory disability scale is insufficient to reflect severity and clinical course of the disease. Standardized systems such as mini-mental state examination, Spastic Paraplegia Rating Scale (67) and Scale for the Assessment and Rating of Ataxia (68) are more appropriate for the purpose.

In conclusion, spastic paraparesis combined with cerebellar involvement is the phenotype of a group of genetically heterogeneous diseases, which are traditionally referred to as HSP, HCA and other inherited neurodegenerative conditions. Recognition of inheritance mode and distinct clinical and neuroimaging features and incorporation of population-specific genetic information may help to guide efficient molecular testing and facilitate genotype-phenotype correlation. Improvement in knowledge regarding biological functions shared by these ASSD genes might open the way for therapeutic approaches targeting a handful of dysregulated cellular pathways instead of single genes or disease symptoms.

## Data availability statement

The datasets presented in this study can be found in online repositories. The names of the repository/repositories and accession number(s) can be found below: https://figshare.com/articles/dataset/Clinico-genetic_characterization_of_spastic_ataxia/20392284.

## Ethics statement

The studies involving human participants were reviewed and approved by Chang Gung Memorial Hospital Institutional Review Board. Written informed consent to participate in this study was provided by the participants' legal guardian/next of kin.

## Author contributions

M-YL, C-SL, and Y-YC: project conceptualization and design. M-YL, C-SL, S-LW, Y-FS, Y-FC, and M-CT: data collection. M-YL and Y-YC: methodology. M-YL and C-SL: genetic testing and data analysis. Y-FC: figure and image editing. M-YL: writing of the first draft. C-SL and Y-YC: manuscript review and critique. All authors contributed to the article and approved the submitted version.

## Conflict of interest

The authors declare that the research was conducted in the absence of any commercial or financial relationships that could be construed as a potential conflict of interest.

## Publisher's note

All claims expressed in this article are solely those of the authors and do not necessarily represent those of their affiliated organizations, or those of the publisher, the editors and the reviewers. Any product that may be evaluated in this article, or claim that may be made by its manufacturer, is not guaranteed or endorsed by the publisher.
