# Supplementary figures and images for: Clinical and genetic characterization of a Taiwanese cohort with spastic paraparesis combined with cerebellar involvement
Source: Front Neurol. 2022 Sep 30;13:1005670. doi: 10.3389/fneur.2022.1005670 (PMC9563621; doi:10.3389/fneur.2022.1005670)

Patient R2

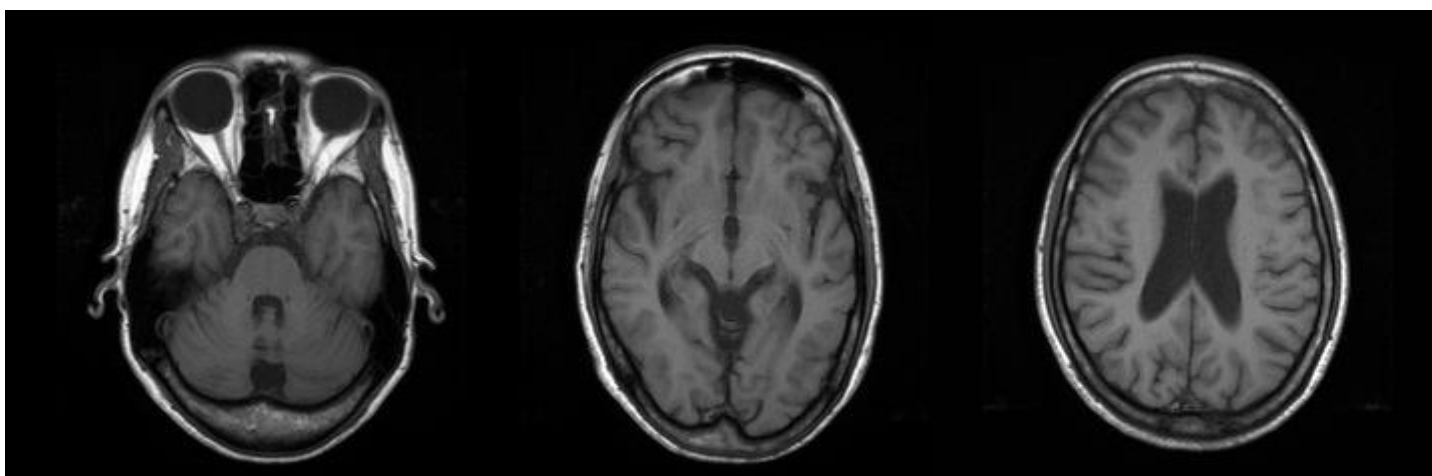

Patient R3-2

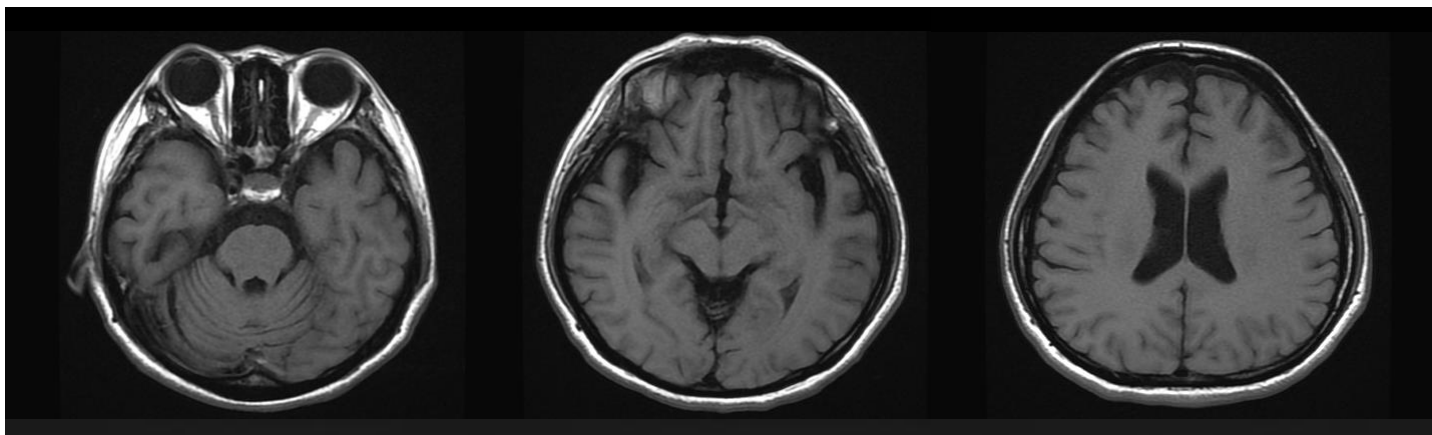

Patient S10

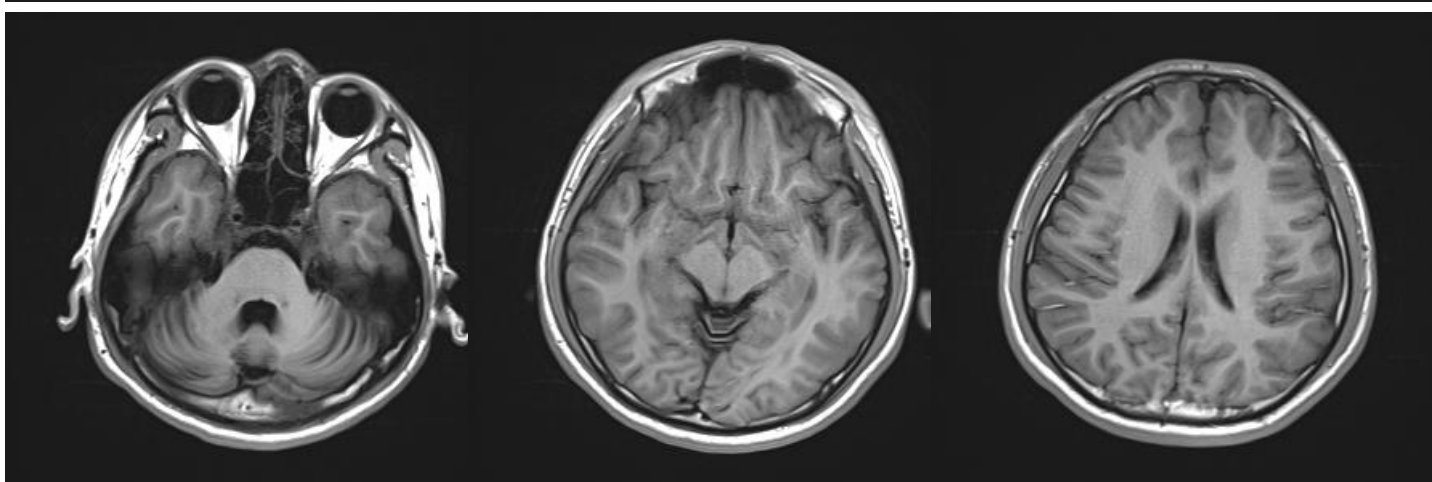

Supplement: Supplementary file 1 [file Data_Sheet_1.PDF]
