# Supplementary material for: Clinical and genetic characterization of a Taiwanese cohort with spastic paraparesis combined with cerebellar involvement
Source: Front Neurol. 2022 Sep 30;13:1005670. doi: 10.3389/fneur.2022.1005670 (PMC9563621; doi:10.3389/fneur.2022.1005670)
Supplement: Supplementary file 2 [file Data_Sheet_2.PDF]

Supplementary Figure

(A)

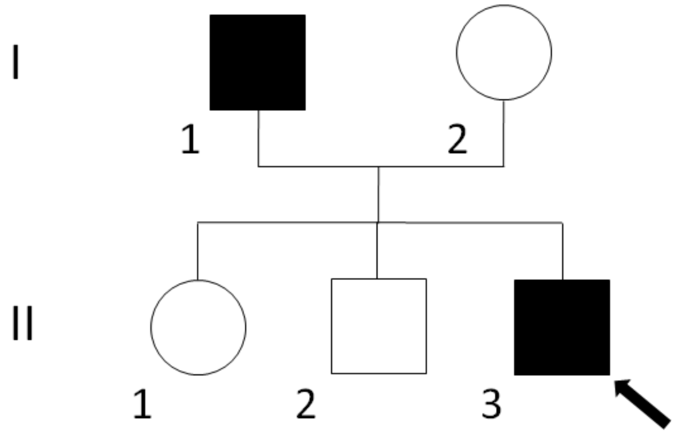

(B)

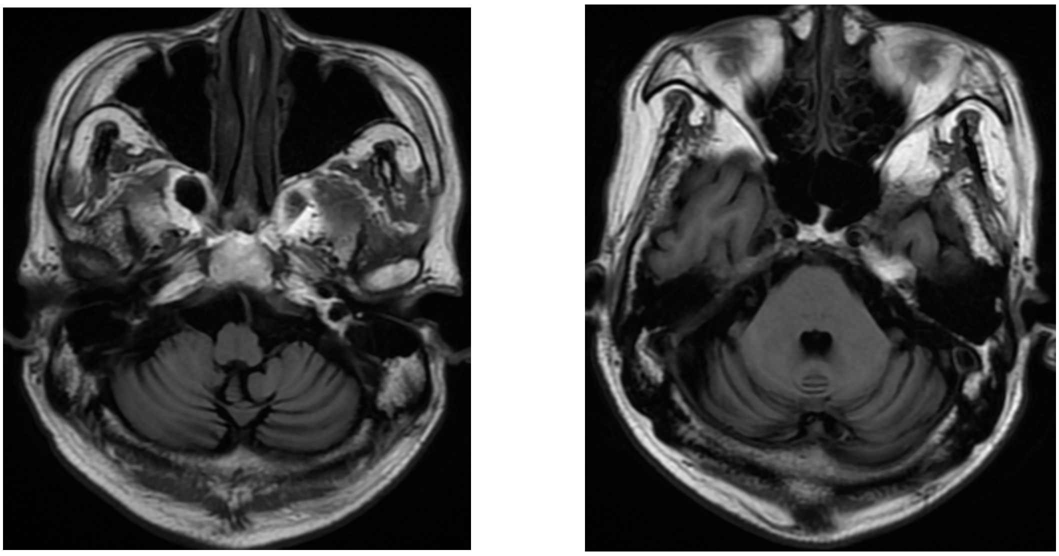

(A) Family D4 pedigree. Proband is indicated by arrow. Squares represent male subjects, and circles represent female subjects. Affected individuals are shown with solid symbols.

(B) Representative T1 weighted brain MR images of case I-1. Diffuse cerebellar atrophy is demonstrated.
